# Comparison of the Efficacy and Safety of Dexmedetomidine Administered in Two Different Modes Under Procedural Sedation and Analgesia in Plastic Surgery

**DOI:** 10.3389/fsurg.2022.836398

**Published:** 2022-05-02

**Authors:** Weipeng Xia, Shanshan Wang, Lingxin Wei, Xiaoming Deng, Dong Yang, Jinghu Sui, Juhui Liu

**Affiliations:** ^1^ Department of Anesthesiology, Plastic Surgery Hospital, Chinese Academy of Medical Science & Peking Union Medical College, Beijing, China; ^2^ Department of Anesthesiology, Beijing Tongren Hospital, Capital Medical University, Beijing, China

**Keywords:** dexmedetomidine, procedural sedation and analgesia, loading dose, hemodynamics, respiration

## Abstract

**Background:**

Dexmedetomidine (DEX), a highly selective α2-adrenergic receptor agonist, is now widely used in procedural sedation and analgesia. This study was designed to observe and compare the efficacy and safety of DEX administered in two different modes.

**Methods:**

In total, 100 patients were randomly divided into two groups to receive intravenous DEX 1 µg/kg over 15 min followed by 0.4–0.7 µg/kg/h infusion or DEX 1 µg/kg over 30 min followed by 0.4–0.7 µg/kg/h infusion. Heart rate (HR), mean arterial pressure (MAP), respiratory rate (RR), bispectral index (BIS), Ramsay Sedation Scores (RSS scores), the lowest respiratory rates (LRR), incidences of respiratory adverse events and frequencies of body movements were recorded. Recovery time, recall of intraoperative events, pain scores in PACU and satisfaction of patients and surgeons were assessed.

**Results:**

The BIS at time points from 5 min after anesthesia to the end of surgery in the intervention group were significantly higher (*p* < 0.05). The RSS scores at time points from 5 min after anesthesia to immediately after induction with DEX were significantly higher in the intervention group (*p* < 0.05). The HR at time points from the beginning of surgery to 30 min after local anesthesia, the MAP at time points from 30 min after local anesthesia to the end of surgery, and the RR at time points from 5 min after anesthesia to the end of surgery were significantly higher in the intervention group (*p* < 0.05). Patients in the intervention group had higher LRR, lower incidences of respiratory adverse events, and shorter recovery time (*p* < 0.05).

**Conclusions:**

Dexmedetomidine infused with a loading dose over 30 min had less impact on patients’ hemodynamics and respiration and could shorten the recovery time after anesthesia in procedural sedation and analgesia.

**Clinical Trial Registration:**

ClinicalTrials.gov, identifier: ChiCTR1900027958.

## Introduction

Dexmedetomidine (DEX) has been used extensively in various superficial surgical procedures and sedation radiographic tests as it induces sedation, analgesia and anxiolysis without significant respiratory depression (1). DEX is currently administered as a loading dose over 10–15 min to rapidly achieve a certain blood concentration, followed by a continuous infusion (2). Due to the limited strength of its sedative and analgesic effects, DEX is often administered to patients with other analgesics or narcotic drugs in sedative and analgesic anesthesia. When used in combination, DEX can improve the anesthetic effect, but may also act to increase the risk of intraoperative respiratory depression (3). Combination of DEX with remifentanil can induce cardiovascular complications such as a lower heart rate (HR) (4).

Previous research reported that omitting the loading dose of DEX avoided hemodynamic side effects without compromising sedation and analgesia (5). No investigation has yet to evaluate whether the prolonged loading dose time of DEX would produce comparable anesthetic effect and decrease the incidences of side effects. With an emphasis on the quality of anesthesia, we designed this study to determine whether the difference in loading dose infusion time would produce different efficacy and safety in procedural sedation and analgesia anesthesia during plastic surgeries.

## Materials and Methods

### Patients

This randomized controlled trial was approved by Ethic Committee of Plastic Surgery Hospital, Chinese Academy of Medical Science (2016–12) and registered in the Chinese clinical trial registry (ChiCTR1900027958). A total of 100 patients from December of 2019 to December of 2020, American Society of Anesthesiologists (ASA) physical status class I or II, aged 18–60 years, and scheduled for elective plastic surgery under procedural sedation and analgesia were enrolled in this trial. The patients were allocated randomly to one of the two groups (*n* = 50) using a computer-generated list of random numbers. Exclusion criteria were: bradycardia (HR < 50 beat/min), hypotension (Mean arterial pressure [MAP] <60 mmHg), severe disease (heart, pulmonary, hepatic or renal), obesity (Body mass index [BMI] ≥30), obstructive sleep apnea/hypopnea syndrome, and known allergy to DEX.

### Monitoring Indicators

No premedication was given to any of the patients. After being taken to the operating room, standard monitoring included electrocardiogram (ECG), noninvasive blood pressure (BP), HR and oxyhemoglobin saturation (SpO_2_) (Datex-Ohmeda Division, Instrumentarium Corp., Helsinki, Finland). Respiratory rate (RR) was monitored by a nasal end-tidal CO2 cannula. The bispectral index (BIS) was also continuously monitored (BIS Vista, Covidien, Boulder, CO, USA). A peripheral intravenous (IV) catheter was inserted by nurse.

### Anesthesia Management

All patients received an IV of midazolam (0.04 mg/kg) and a continuous infusion of remifentanil (0.1 µg/kg/min) at the beginning of anesthesia. Patients in the control group received IV DEX 1 µg/kg over 15 min followed by 0.4–0.7 µg/kg/h infusion and patients in the intervention group received IV DEX 1 µg/kg over 30 min followed by 0.4–0.7 µg/kg/h infusion. During the surgery, the level of sedation status was assessed using the Ramsay Sedation Scores (RSS) (6) (**Table 1**) and the BIS. The goal of sedation was to achieve an RSS of 3–5 or BIS of 60–80. Therefore, local anesthesia was performed. The patients’ MAP and HR were maintained at a range of baseline ±20%. Atropine (0.3 mg) and ephedrine (6 mg) were intravenously administered for bradycardia (HR < 50 beats/min for >60 s) and hypotension (MAP < 60 mmHg for >60 s), respectively. A facial oxygen mask (6 L/min) was used for hypoxia (SpO_2_ < 90%). If airway obstruction occurred, the patient was treated by thrusting the jaw. Midazolam (2 mg) was administered as a rescue drug when the infusion rate of DEX was increased to a maximum of 0.7 µg/kg/h and the depth of sedation was not achieved. The infusion of DEX was stopped 30 min before the end of surgery, and the infusion of remifentanil was turned off at the end of the surgery.

**Table 1:** Satisfaction scale and ramsay sedation scores.

Satisfaction scale	
0	Extremely dissatisﬁed
1	Dissatisﬁed
2	Neutral
3	Satisﬁed
4	Extremely satisfied
Ramsay Sedation Scores	
1	Anxious and agitated, restless
2	Cooperative, oriented, tranquil
3	Responsive to verbal commands, drowsy
4	Asleep, responsive to light stimulation
5	Asleep, slow response to stimulation
6	No response to stimulation

### Outcome measurements

HR, MAP, RR, BIS, RSS were evaluated and recorded before anesthesia (baseline) (T_0_), 5 min after anesthesia (T_1_), 10 min after anesthesia (T_2_), immediately after induction with DEX (T_3_), the beginning of local anesthesia (T_4_), the beginning of surgery (T_5_), 30 min after local anesthesia (T_6_), 60 min after local anesthesia (T_7_), immediately after turning off DEX infusion (T_8_), and the end of surgery (T_9_). The lowest respiratory rates (LRR), incidences of respiratory depression (RR < 10 breaths/min), incidences of oxygen supplementation by facial mask and thrusting the jaw, frequencies of body movements, and additional rescue drug administrations were also recorded throughout the procedure. After surgery, recovery time, recall of intraoperative events, and pain scores using a numerical pain scale (0 = no pain to 10 = the worst pain) (7) in PACU were assessed. The satisfaction of patients and surgeons with the anesthesia were assessed using a 5-point numerical rating scale (8) (**Table 1**).

### Statistical Analysis

All statistical analyses were performed using SPSS 23.0 (SPSSFW, SPSS Inc.). The normality of distribution was assessed with the Shapiro-Wilk test. Normally distributed data are presented as mean ± SD, and skewed data were presented as a median with an interquartile range [IQR]). Independent t-test was used to examine the difference of normally distributed continuous variables between groups. Skewed data were analyzed by Mann-Whitney U-test. Categorical data are presented by frequency and analyzed by chi-square test. A *p* value of <0.05 was considered statistically signiﬁcant.

## Results

**Figure 1:**
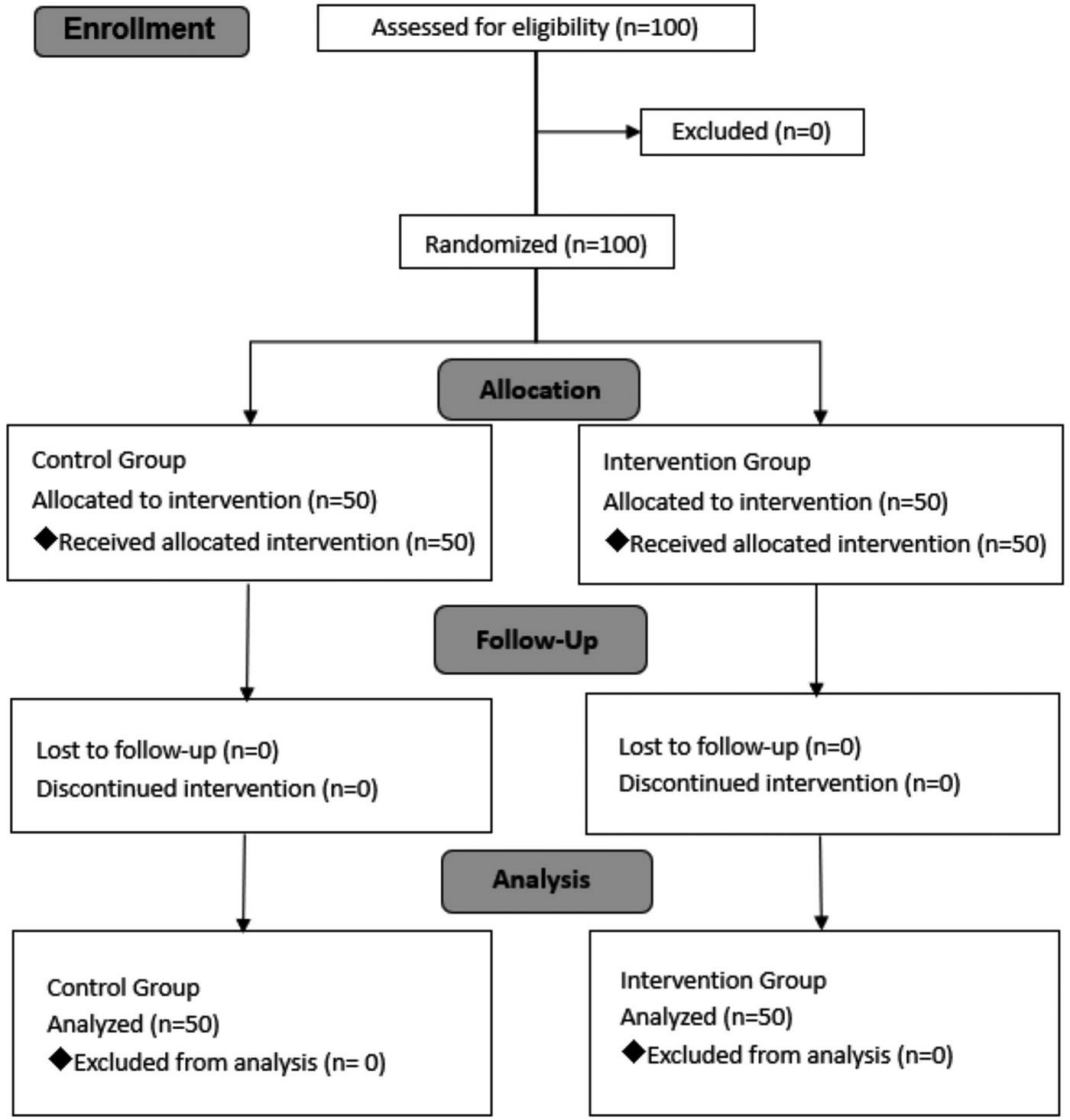
CONSORT Flow Diagram.

The procedure was successfully performed in each of the 100 patients recruited in the study (**Figure 1**). No significant differences were observed in patient demographic data including gender, age, ASA classification and BMI (**Table 2**). The surgery time did not differ between groups.

**Table 2:** Patients’ demographic data and surgery time.

	Control group	Intervention group	*p* value
Gender (M/F)^a^	8/42	6/44	0.564
Age (yrs)^b^	31.7 ± 8.2	30.3 ± 6.1	0.336
BMI (kg/m^2^)^b^	21.6 ± 2.6	21.7 ± 2.6	0.098
ASA (I/II)^a^	30/0	30/0	1.000
Surgery time (min)^b^	123.30 ± 37.6	126.1 ± 44.9	0.740

*Data are expressed as the mean ± SD, or number (%). BMI (Body mass index) = weight/(height)^2^*

*^a^* *Chi-Square Test.*

*^b^independent sample t-test*.

**Figure 2:**
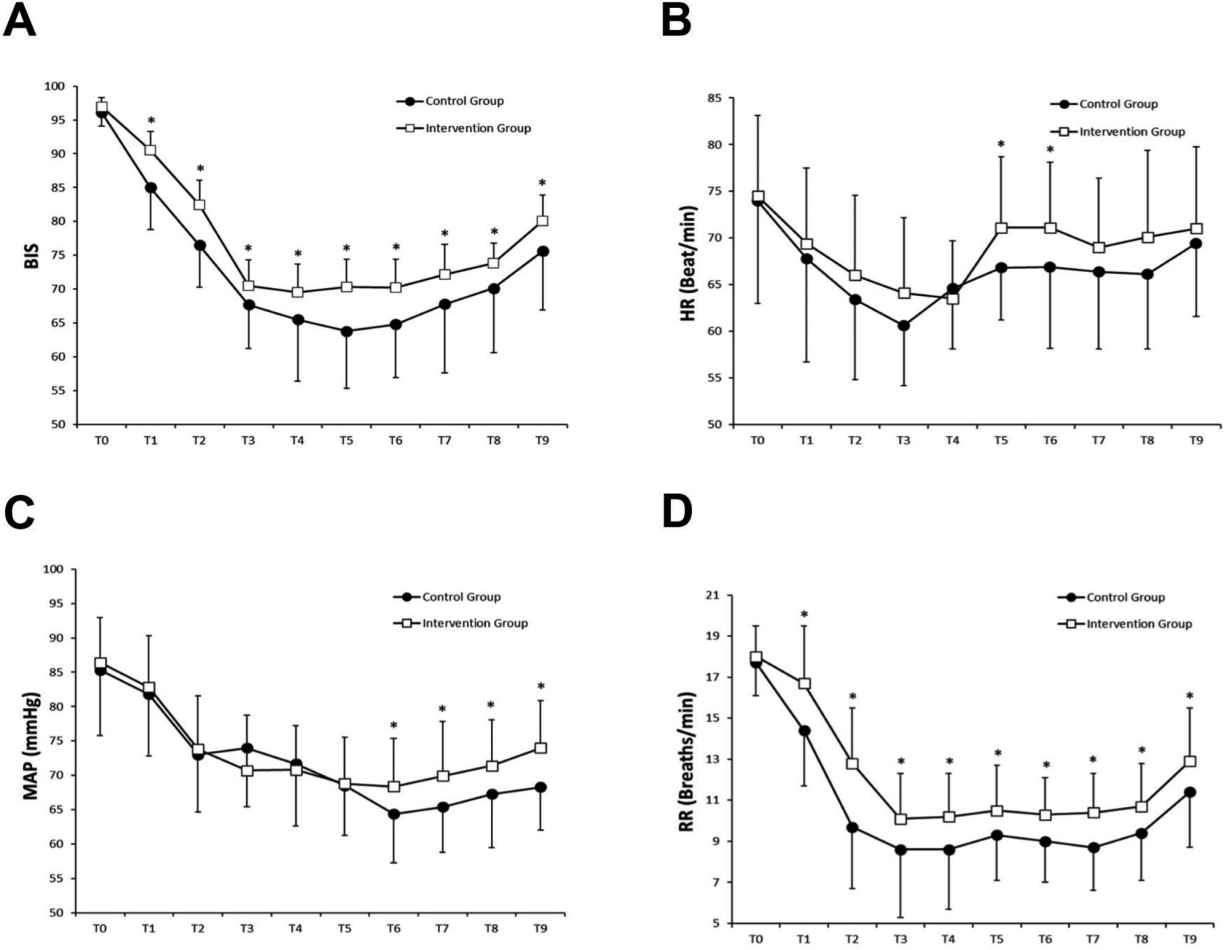
Changes in BIS (**A**), HR (**B**), MAP (**C**) and RR (**D**) were compared by the independent sample t-test for comparisons between the two groups. *p < 0.05 compared with the Control group.

**Figure 2A** and **Table 3** show the changes in sedation level during the study. Patients in the intervention group had higher BIS at time points from 5 min after anesthesia to the end of surgery (*p *< 0.05). Patients in the intervention group also had higher RSS scores compared with the control group at time points from 5 min after anesthesia to immediately after induction with DEX (*p *< 0.05).

**Table 3:** Ramsay sedation scores.

	Control group	Intervention group	*p* value
T_0_	2 (2–2)	2 (2–2)	1.000^a^
T_1_	3 (2–3)	2 (2–2)*	0.000^a^
T_2_	4 (3–4)	3 (3–3)*	0.000^a^
T_3_	4 (4–4)	4 (4–4)*	0.006^a^
T_4_	4 (4–4)	4 (4–4)	0.067^a^
T_5_	4 (4–4)	4 (4–4)	0.155^a^
T_6_	4 (4–4)	4 (4–4)	0.242^a^
T_7_	4 (4–4)	4 (4–4)	0.705^a^
T_8_	4 (4–4)	4 (4–4)	0.080^a^
T_9_	4 (4–4)	4 (3–4)	0.295^a^

*Data are expressed as medians (interquartile range).*

*^a^* *Mann-Whitney U Test.*

* *p < 0.05 compared with the Control group.*

**Figure 2B–D** displays the hemodynamic and respiratory parameters changes during the study. Patients in the intervention group had higher HR at time points from the beginning of surgery to 30 min after local anesthesia, higher MAP at time points from 30 min after local anesthesia to the end of surgery, and higher RR at time points from 5 min after anesthesia to the end of surgery (*p *< 0.05).

Compared with the control group, patients in the intervention group had higher LRR, lower incidences of respiratory depression, lower incidences of oxygen supplementation by facial mask and thrusting the jaw, and a shorter recovery time (*p *< 0.05) (**Table 4**).

**Table 4:** Assessments of intraoperative events and evaluations in PACU.

	Control group	Intervention group	*p* value
Lowest respiratory rate (breaths/min)^a^	6.9 ± 2.4	8.6 ± 1.9*	0.000
Respiratory depression^b^	40 (80.0)	25 (50.0)*	0.002
Incidences of oxygen supplementation by facial mask^b^	40 (80.0)	13 (26.0)*	0.000
thrusting the jaw^b^	17 (34.0)	2 (4.0)*	0.000
recall of events during surgery^b^	0 (0)	0 (0)	1.000
Frequencies of body movements^b^	0 (0–0)	0 (0–0)	0.781
additional rescue drug administrations^c^	1 (0.75–2)	1 (0–2)	0.621
Recovery time (min)^a^	22.9 ± 8.8	16.0 ± 2.9*	0.000
VAS score^c^	0 (0–1)	0 (0–1)	0.171
Satisfaction score of patients^c^	4 (4–4)	4 (4–4)	0.317
*Satisfaction score of surgeons* *^c^*	*4* *(4–4)*	*4* *(4–4)*	*0* *.* *317*

*Data are expressed as the mean ± SD, median (interquartile range), or number (%).*

*^a^* *independent sample t-test.*

*^b^* *Chi-Square Test.*

*^c^* *Mann-Whitney U Test.*

* *p < 0.05 compared with the Control group.*

No significant differences were found in body movements, additional rescue drug administrations, recall of intraoperative events, pain scores and the satisfaction levels of patients and surgeons between groups (*p *> 0.05) (**Table 4**).

## Discussion

Due to its numerous advantages, DEX is one of the main anesthetics currently used for procedural sedation and analgesia. In this study, we found that although DEX infused with a loading dose over 15 min provided deeper sedation than DEX infused with a loading dose over 30 min, these two different DEX administrations could both be effectively applied in the surgery. In addition, DEX infused with a loading dose over 30 min resulted in more stable hemodynamics, less respiratory depression, and shorted recovery time for patients in procedural sedative and analgesic anesthesia.

In order to achieve satisfactory sedation and analgesia, DEX is often used clinically in combination with other anesthetic drugs such as midazolam and opioids (9–11). Midazolam reduces the incidence of recall of intraoperative events by producing anterograde amnesia (12). Studies have shown that DEX combined with midazolam provided better sedation than midazolam alone in patients undergoing fiberoptic bronchoscopy (13). The analgesic effect of DEX combined with opioids is beneficial in invasive operations. For example, a previous study demonstrated dexmedetomidine combined with remifentanil provided better analgesia and higher satisfaction levels amongst surgeons than midazolam combined with remifentanil in patients undergoing radiofrequency ablation for atrial fibrillation (8). In our study, DEX was administered with midazolam and remifentanil to produce sufficient sedation and analgesia for the surgery.

While enhancing the anesthetic effect, the combination of sedation and analgesic drugs may also increase the incidences of respiratory and circulatory depressions. Clinically, the safety of anesthesia can be improved by reducing the dose of drugs or adjusting the method of drug administration (14, 15). In our study, we extended the infusion time of DEX loading dose from 15 to 30 min to delay the increment of DEX plasma concentration and attenuate the DEX peak plasma concentration. As shown in the present study, patients in the intervention group had a more gradual BIS change after DEX infusion. However, no differences were found between the groups in RSS from the beginning of local anesthesia to the end of surgery, intraoperative body movements, additional rescue drug administrations, recall of intraoperative events, pain scores in PACU, or the satisfaction levels of patients and surgeons. Our results revealed that although the sedation depth in the intervention group was relatively shallow, this mode of DEX administration could achieve the sedation and analgesia required for the surgery. In a study of sedative and analgesic anesthesia, use of dexmedetomidine could significantly prolong postoperative recovery time (16). In this study, the DEX plasma concentration of the intervention group after the loading dose infusion was theoretically lower than that of the control group. We found no differences in the surgery time between groups which lead to a better early postoperative recovery for patients in the intervention group.

With respect to hemodynamic stability, Bloor *et al*. (17) reported that the amount of DEX loading dose and the duration of administration affected the blood pressure, with blunting of the blood pressure by 23% 60 min after infusion of 1 µg/kg DEX over 2 min. The remifentanil used in this study is a non-accumulative, ultra-short-acting opioid which allows faster recovery after anesthesia (18, 19). We know from earlier studies that both remifentanil and DEX reduce HR (20, 21). We observed that the intervention group had higher MAP at time points from 30 min after local anesthesia to the end of surgery and higher HR at time points from the beginning of surgery to 30 min after local anesthesia, which indicated that prolonged DEX loading dose time contributed to more stable hemodynamics.

DEX is seldom administered in clinical practice as a single agent for procedural sedation and analgesia. It is often administered in combination with analgesics. Though DEX provides sedation and analgesia without significant respiratory depression, respiratory changes needed to be measured in the case of drug combinations (22). The depth of sedation can significantly affect patients’ minute ventilation and RR (23). In the present study, we found both groups showed respiratory depression during anesthesia. However, the intervention group had higher RR throughout the study and also had higher LRR, lower incidences of respiratory depression, oxygen supplementation by facial mask and thrusting the jaw. We concluded that prolonged DEX loading dose time reduced adverse respiratory reactions.

## Limitations

There are a few limitations to this study. The method of DEX administration in the control group is extensively applied in clinical practice. Though higher respiratory depression was observed in this group, the problem could be solved by oxygen supplementation with facial mask or thrusting the jaw. Before the study, we excluded patients with known obesity or obstructive sleep apnea/hypopnea syndrome. For patients with a predictable risk of respiratory depression, further research is needed to identify the safety of this method of DEX administration.

## Conclusions

In conclusion, our study demonstrated that DEX is an excellent drug when combined with other narcotic drugs in sedative and analgesic anesthesia. Prolonging the DEX loading dose infusion time from 15 to 30 min can provide sufficient sedation during the surgery with more stable hemodynamics, as well as less respiratory adverse reactions.

## Data Availability

The raw data supporting the conclusions of this article will be made available by the authors, without undue reservation.
